# Compliance with health protocols in the banking sector facing Covid-19

**DOI:** 10.3389/fpubh.2023.1129578

**Published:** 2023-06-27

**Authors:** Leila Ibrahimi Ghavamabadi, Mitra Shabab, Behzad Fouladi Dehaghi, Farkhondeh Hoveizi

**Affiliations:** ^1^Department of Environmental Management-HSE, Ahvaz Branch, Islamic Azad University, Ahvaz, Iran; ^2^Environmental Technologies Research Center, School of Public Health, Ahvaz Jundishapur University of Medical Sciences, Ahvaz, Iran; ^3^Department of Occupational Health Engineering, School of Public Health, Ahvaz Jundishapur University of Medical Sciences, Ahvaz, Iran

**Keywords:** COVID-19, bank, staff, health, prevention, protocol

## Abstract

**Background:**

The spread of the coronavirus has become one of the current challenges in the world. Implementing health protocols in the workplace along with informing people who face them, has become a vital issue.

**Objective:**

The purpose of this study was to determine the level of compliance with health protocols and measure the level of awareness, attitude and performance of bank employees while facing Covid-19.

**Materials and methods:**

The data collection tool included a checklist and two questionnaires (demographic information and the other on the awareness, attitude and performance of bank staff) regarding the coronavirus.

**Results:**

A total of 199 bank employees from 25 bank branches participated in the study. The results showed that in 24% of the bank branches, the staff did not use face masks. And 100% of the studied bank branches lacked proper ventilation. The mean awareness scores of female employees (94.3) were significantly higher than those of male employees (87.7). Also, the performance of female employees (93.2) was significantly better than male employees (87.2).

**Conclusion:**

The results showed that to achieve proper performance in controlling and reducing the covid-19 disease among bank employees and other workers, these items are noteworthy: (1) Providing and training the correct use of protective equipment including face masks. (2) Monitoring for strict implementation of health protocols. Therefore, it is necessary to keep training and information up-to-date due to the new phenomena of this disease.

## 1. Introduction

The Covid-19 pandemic with the new SARS-CoV-2 coronavirus was observed in late 2019 in the city of Wuhan-China, and the world health organization announced a pandemic in the world (1). The reported symptoms of Covid-19 include respiratory symptoms with acute respiratory distress syndrome, which ultimately leads to patient death in the most severe cases (1). In some previous studies in the field of SARS or Ebola epidemic diseases, the occurrence of a sudden and life-threatening disease can put a lot of pressure on public service employees such as medical staff (2). In the banking sector, the risks of COVID-19 increase concerns about the health and safety of bank employees and customers, in order to protect the health of employees to provide service and the risks of liability related to contracting the virus in the bank environment (3). Moreover, In the bank environment, due to the high number of customers, limited space, insufficient ventilation, common contact surfaces and long working hours, the conditions for the transmission of airborne viral diseases are provided (4). Also, many studies stated that bank staff are exposed to a variety of occupational stressors and illnesses, which can lead to serious physiological and psychological harm (4–6). Increased workload, physical exhaustion, inappropriate personal equipment and disease transmission may have significant effects on the physical and mental health of employees (7, 8). Therefore, these employees are particularly vulnerable to mental health problems, including fear, anxiety, depression, and insomnia (9). Anxiety, stress and mental concerns of a person due to the society’s view of the sick person are among the mental health disorders that exist in all diseases and biological disorders (6, 10, 11). Bank employees are in daily contact with a large number of customers due to their job conditions and as a result their increase the level of stress and anxiety in such individuals (4, 6). Establishing clear risk communication, limiting working hours, as well as providing access to protective equipment and training on how to cope with the disease of Covid-19 in the workplace can reduce the anxiety caused by the uncontrollability of risks (12, 13). The results of a study conducted by Zhang et al., to investigate the level of knowledge and attitude of employees, including health workers, in the outbreak of the new coronavirus, showed that 89% of all individuals had sufficient knowledge about Covid-19 (14). Preventing transmission of Covid-19 requires awareness and individual willingness to adhere to preventive measures (15). Therefore, surface disinfection, frequent hand washing, physical distancing and use of personal protective equipment (PPE) are recommended preventive measures (16). Also, the results of several studies stated that people’s performance in facing the corona disease can be affected by factors including the availability of information sources, awareness, attitude and socio-demographic characteristics (17–19). Moreover, according to the report by Iran’s central bank, 260 bank employees have died of corona virus infection in the first months of pandemic in Iran in 2020 (20). Considering the above-mentioned cases and the higher risks of contracting diseases in bank employees who are in close contact with customers, it is necessary to check the knowledge of bank staff in terms of ensuring their health and individual functioning in the organization, and it will allow to plan about their working conditions and, if necessary, to develop suitable training programs. Therefore, the purpose of this study was to determine the level of compliance with health protocols and to compare the level of awareness, attitude and performance of bank employees while facing Covid-19.

## 2. Materials and methods

### 2.1. Study design and participants

The present study was a cross-sectional descriptive study performed in Ahvaz in Iran during April to August 2021. The G power software was used to calculate the sample size. The alpha error and confidence level were assumed at 5 and 90%, respectively. The calculated sample size was 199.

### 2.2. Instrument

The data collection tool included a checklist and two demographic information questionnaires and a questionnaire on the knowledge, attitude and performance of bank personnel regarding the coronavirus. The criteria for entering the study include responding to at least 5 bank customers during working hours. Exclusion criteria included incomplete questionnaires and unwillingness to complete the questionnaires. To check the health status and how to comply with the instructions of the national corona committee, the written checklist of the ministry of health of Iran was used. The checklist items included the correct use of face masks, compliance with physical distance, access to disinfectants in the workplace, the condition of the ventilation system, and use of shared equipment by clients. The demographic information questionnaire included age, gender, level of education, work experience and history of corona disease. Five questions were also asked regarding coronavirus anxiety. These 5 questions were in the form of a five-point Likert scale (strongly disagree, disagree, undecided, agree and strongly agree). Scoring was done as strongly disagree = 0, disagree = 1, undecided = 2, agree = 3 and strongly agree = 4. The minimum and maximum scoring were between zero and 20. The questionnaire regarding awareness, attitude and performance of the bank staff regarding the coronavirus had eight questions in the field of awareness of the coronavirus, recognition of the symptoms of the disease, knowledge on ways of transmission of the disease, groups at risk and diagnosis and preventive measures. General awareness of coronavirus included three questions. In order to check the level of awareness of the symptoms of the disease, questions were asked about the symptoms of the disease such as fever, cough, sore throat, body ache and headache. These questions were answered by yes/no and I do not know. In order to check the level of knowledge about the ways of disease transmission, a four-choice question was considered where the participants were free to choose more than one option. To select the groups at risk, a five-choice question was considered, and the participants could choose more than one options. A positive score was given to each of the option of older adult people, pregnant women and people with a weak immune system and underlying disease (cancer, chronic respiratory diseases, diabetes, kidney disease) and zero were assigned to the “I do not know” option. Four questions were asked in the diagnosis and preventive measures section. These questions were answered by yes/no and I do not know. The attitude scale included 14 questions on a three-point Likert scale of yes/no, and I do not know, where the “yes” option was scored with a score of 1, and the “no” and “I do not know” options were scored with a score of zero. The minimum and maximum scoring in this part was between zero and 14. The performance part also included 10 questions, seven of which were on a three-point Likert scale of yes / no, and I do not know, and the “yes” option was scored with a score of 1, and the “no” and “I do not know” options were scored with a score of zero. The minimum and maximum scoring in these 10 questions were between 0 and 10. A question was also devoted to the use of masks. This question consisted of four options (I do not use it, only in crowded places and gatherings, most places and always). Checking the risk of contracting the coronavirus also included two questions on a Likert scale with options ranging from very little to very much. The scope of the source of the received information and the level of trust in media also included two questions on a five-point Likert scale with options from very little to very much, which were scored from 1 to 5. The minimum and maximum scoring in this part were between 1 and 5.

### 2.3. Reliability and validity

The reliability of the questionnaire was determined by determining the Cronbach’s alpha coefficient for the variables of awareness 0.72, attitude 0.72, performance 0.82, source of information 0.75 and trust in the media 0.72, respectively. Also, the validity of the questionnaire was determined and confirmed by using the opinions of five faculty members of the university.

### 2.4. Statistical analysis

Data analysis was carried out using the SPSS software version 21 and the descriptive statistical tests such as frequency, percentage, independent *t*-test and one-way analysis of variance were used to compare the average scores of awareness, attitude and performance of bank staff at a significance level of *p* < 0.05 were considered.

## 3. Results

A total of 199 individuals (19.6% women and 80.4% men) from the 25 bank branches participated voluntarily in this study. Table 1 presents the demographic information of the participants. According to the results of the polymerase chain reaction (PCR) test, 49 of the employees were confirmed as positive Covid-19 cases.

**Table 1:** Demographic characteristics of participants.

Characteristic	Male group (*n* = 160)	Female group (*n* = 39)
*Age range (year)*		
30–34	50 (31.2)	12 (30.7)
35–39	88 (50.6)	21 (53.8)
>40	29 (18.1)	6 (15.3)
*Job experience (year)*		
<10	70 (43.7)	18 (46.1)
11–20	60 (37.5)	18 (46.1)
>21	30 (18.7)	3 (7.6)
*Infected by COVID-19*		
Yes	42 (26.2)	7 (17.9)
No	118(73.7)	32 (82.1)
*Education*		
Secondary school	20 (12.5)	0 (0.0)
Technical training	45 (28.1)	2 (5.2)
University	95 (59.3)	37 (94.8)

Values are presented as number (%).

The results of the preliminary investigation according to the checklist of the ministry of health showed that in 24% of the bank branches, the staff did not use face masks. And in 100% of the studied bank branches, the customers used commonly shared devices such as pens and stamps. 88% of the bank branches had a manual queuing machine and 12% of the bank branches had a foot pedal queuing machine. 100% of the studied bank branches lacked proper ventilation, 80% of the bank branches lacked disinfectants for customers to use, and 24% of the bank branches lacked disinfectants for employees to use. 84% of the bank branches did not have any control measure at the entrance of banks to prevent the entry of customers suspected of Covid-19. Maintaining physical distance was observed by customers and employees in 28 and 32% of the bank branches, respectively. Table 2 showed the bank staff’s responses to anxiety questions. According to the results, most of the answers given to each item were similar in both groups. The results of the awareness and general information about Covid-19 were presented in Tables 3, 4. According to the results, most of the participants answered the questions correctly (it shows a high level of awareness in the participants). Also, regarding taking necessary measures in case of suspected symptoms of corona virus disease in themselves or others, 97% of female employees and 89% of male employees stated that they measure the degree of fever. And 98% of female employees and 95% of male employees refer to a doctor. Also, 82.3% of female employees and 80.3% of male employees stated that they will avoid doing normal and daily activities. In contact with suspicious people, 87% of male employees and 100% of female employees avoid suspicious people. Table 5 shows the results of the questions related to the participants’ attitude measurement section. According to the results regarding attitude questions, most of the individuals’ views were in line with general recommendations of disease prevention.

**Table 2:** The percentage of participants’ response to the anxiety questions.

	Groups	Strongly disagree	Disagree	Undecided	Agree	Strongly agree
I am afraid of getting corona virus	Male	5.1	24	7.9	42	21
	Female	4	8	6	39.5	42.5
I avoid going to public places because of the fear of Corona virus	Male	2	16	2	39.2	40.8
	Female	0	3.9	7.2	41.7	47.2
I do not touch common surfaces because of the fear of Corona	Male	2.3	4.2	5.1	52.4	36
	Female	0	0	4.3	43.5	52.2
Because of my fear of corona, I will die if I get infected	Male	6.4	22.8	24.1	40.5	6.2
	Female	4.2	12.7	19.7	37.2	26.2
There is a high probability that I will get corona virus	Male	3.3	8.7	25.1	46.5	16.4
	Female	3.5	5.5	7.7	33.5	49.8

**Table 3:** The results of the level of awareness of the corona disease in the participants.

Question	Answer	Male	Female
Has anyone in your family or acquaintances been infected with corona disease?	Yes	86%	86.2%
	No	11.5%	11.8%
	I do not know	2.5%	2%
Is corona disease contagious?	Yes	93%	98%
	No	5	0
	I do not know	2	2
Which case is the cure for Corona?	Treatment based on the symptoms of the disease	25%	16%
	Antibiotics	5	6
	There is no cure	60	64
	I do not know	10	14
In your opinion, the symptoms of the disease appear a few days after infection.	< 2 days	4%	3%
	2–5 days	22	34
	3–14 days	43	53
	I do not know	31	10
At what age is this disease more dangerous?	30 to 50 year	24	35
	>50	76	65

**Table 4:** The results of the general information of the participants regarding the corona disease.

Disease Symptoms	Diarrhea		Body pain		Headache		Sore throat		Cough		Fever	
	Male	Female	Male	Female	Male	Female	Male	Female	Male	Female	Male	Female
Yes	79%	96%	97%	99%	76%	91%	89%	95%	95%	97%	89%	97%
No	10%	2	3	1	13	8	6	4	3	3	2	1
I do not know	11	2	0	0	11	1	5	1	2	0	9	2

Disease transmission	Through coughing		Through surfaces		Through food		Through people	
	Male	Female	Male	Female	Male	Female	Male	Female
Yes	96	97.5	92	93.5	33	30	86	98
No	4	2.5	8	6.5	67	70	14	2

Groups at risk	Older adult		Pregnant women		People with immune system deficiency		People with underlying disease	
	Male	Female	Male	Female	Male	Female	Male	Female
Yes	95	100	93	100	89	97	100	100
No	5	0	7	0	11	3	0	0

**Table 5:** The results of attitude to the disease.

Question	Answer	Male	Female	Question	Answer	Male	Female
Does early diagnosis lead to improved treatment?	Yes	92.5%	95.2%	If you are careful, can you prevent corona disease?	Yes	95%	93%
	No	5.3	3.5		No	4	4
	I do not know	2.2	1.3		I do not know	1	3
Can corona disease be treated at home?	Yes	79.5	75.4	Can the corona disease be transmitted through pets?	Yes	26.5	28.5
	No	18.5	22.6		No	51	52
	I do not know	2	2		I do not know	22.5	19.5
Is corona disease dangerous?	Yes	85	88	Is the prevalence of disease increasing in the country?	Yes	86	88
	No	9	7		No	4	3.5
	I do not know	6	5		I do not know	10	8.5
Do you use vaccines?	Yes	87	90	Does washing hands with soap lead to the elimination of the disease agent?	Yes	96.2	97.5
	No	11	8		No	2	1.5
	I do not know	2	2		I do not know	1.8	1
Can corona disease be cured?	Yes	65	67	Should travel be limited to prevent the spread of the disease?	Yes	88	92
	No	31	31		No	12	8
	I do not know	4	2		I do not know	0	0
Is it enough to inform about the corona disease through the media?	Yes	60	60	Should patients be quarantined in the hospital?	Yes	91	93
	No	29	27		No	8	6
	I do not know	11	13		I do not know	1	1
Does corona disease lead to death in all cases?	Yes	6.5	7.3	Should the entire community be quarantined to prevent the spread of the disease?	Yes	85	88
	No	91	89		No	12	10
	I do not know	2.5	3.7		I do not know	3	2

Also, the overwhelming majority of the participants agreed to create more restrictions to prevent the spread of the disease. In most of the questions, the level of agreement with creating restrictions was higher among female employees. In this regard, 94.9% of male employees and 100% of female employees were in favor of restricting travel to high-risk areas, 93% of female employees and 91% of male employees were in favor of quarantining infected patients in special hospitals, 100% of female employees and 98% of male employees were in favor of closing educational centers (kindergartens, schools and universities). In line with disease prevention, the performance of most personnel was evaluated positively (Table 6). According to the results, the actions of most of the participants were as follows: 82% of male employees and 57% of female employees did not leave home and 75% of the two groups avoided unnecessary travel. Regarding the use of herbal products and traditional medicine, the rate of use by female employees was higher, so 96% of female employees and 88% of male employees stated that they use these products. But in the case of taking vitamin supplements, the rate of use was similarly reported in both groups. According to the results, in both groups of employees, the performance of women was significantly higher than that of men (*p* < 0.05). In male employees, the average attitude score of participants who had no history of traveling during the pandemic was significantly higher than others (*p* = 0.021). In male participants, there was a significant difference in all three variables of awareness, attitude and performance between the staff with different educational levels (*p* < 0.05), so with a higher level of education, the score of these variables also increased. In other cases, no significant difference was observed between the average scores of awareness, attitude and performance of individuals in both groups based on demographic variables (*p* > 0.05). In order to compare the awareness, attitude and performance of employees, an independent t-test was used. According to the obtained results, the average awareness scores of female employees (with an average of 94.3) were significantly higher than those of male employees (with an average of 87.7; *t* = 9.5, *p* < 0.001). Also, the performance of female employees (with an average of 93.2) was significantly better than male employees (with an average of 87.2; *t* = −2.710, *p* = 0.03). There was no significant difference between the mean attitude scores of the two groups (*p* = 0.43; Table 7).

**Table 6:** Performance questions in order to prevent the disease of Covid-19.

Question	Answer	Male	Female	Question	Answer	Male	Female
I do not leave the house	Yes	82.1%	57%	Attention to regular hand washing and personal hygiene	Yes	86%	98%
	No	14.1	27.4		No	14	2
	I do not know	3.8	15.6		I do not know	0	0
Avoid unnecessary travel	Yes	75	75.4	Regular use of disinfectants	Yes	79	91
	No	10	5.6		No	15	9
	I do not know	15	19		I do not know	6	0
Avoid eating outside	Yes	85	87	Using traditional medicine and herbal medicines to prevent disease	Yes	88	96
	No	10	10		No	0	0
	I do not know	5	3		I do not know	12	4
Avoid Shaking Hands	Yes	89	93	Use of vitamin supplements	Yes	95	95
	No	11	7		No	5	0
	I do not know	0	0		I do not know	0	5
Avoid using public transportation	Yes	78	81	Constant use of face masks	Yes	82	89
	No	20	16		No	18	11
	I do not know	2	3		I do not know	0	0

**Table 7:** Comparison between the mean scores of awareness, attitude and performance in two groups.

Variable	Groups	Mean ± SD	*t*	*p*-value
Awareness	Male	87.75 ± 7.77	5.9	0.001
	Female	94.35 ± 5.27		
Attitude	Male	75.4 ± 13.4	0.787	0.43
	Female	77.9 ± 11.99		
Performance	Male	93.2 ± 12.5	−2.67	0.03
	Female	87.2 ± 14.6		

## 4. Discussion

The present study was conducted with the aim of investigating compliance with health protocols and also comparing the level of awareness, attitude and performance of bank staffs in the face of the Covid-19 virus during the outbreak. Awareness is a necessary base for creating preventive beliefs, forming a positive attitude and promoting positive behaviors. Generally people’s awareness and attitude toward the disease affect the effectiveness of their coping strategies and behaviors to some extent (21). A review of the research background shows that few studies have paid attention to bank employees. Meanwhile, this group of working individuals plays an important role in providing public services to society. The results of the study showed that male employees follow preventive measures less than female employees. This finding has also been stated by Qanche et al. and Kassa et al. in other communities (22, 23), which indicates that women are more committed to preventive measures (24). The findings of the current study showed that people who have a favorable attitude toward COVID-19 are more likely to take good preventive measures against COVID-19. Similar findings are reported by Reuben et al., and Shah et al., (25, 26). The results of a study in the banking sector by Yasmin et al. stated that the level of stress, depression and anxiety in bank staff were high (11). A similar finding was observed in this study regarding anxiety. In another study by Bijani and Jabari the results stated that the level of anxiety about corona virus was related to the level of knowledge (27). This is likely because positive outlooks result in adherence to preventive measures for COVID-19. This means that if a person feels positive about disease prevention, he/she is more likely to follow preventive measures. The study found that only 24% of bank branches meet national requirements related to the prevention of COVID-19. This low level of standards’ compliance is alarming because these institutions which provide public services can act as carriers of disease in society due to the number of clients. Therefore, creating a favorable environment to comply with health regulations leads to strengthening the attitude and performance of people in the face of the Covid-19 disease.

One of the limitations of the present study was the impossibility of directly observing the behavior of the bank staff and collecting information related to the behaviors through self-report, which may cause bias in the evaluation of the results.

## 5. Conclusion

The findings of the study showed that the rate of compliance with health protocols in the examined banking sector was obtained at least at 50 percent in general. Also the awareness, attitude and performance among banking sector employees in the context of the COVID-19 pandemic are influenced by their knowledge. Therefore, this study shows the importance of providing factual knowledge in facing corona disease. In order to achieve proper performance in controlling and reducing the covid-19 disease among bank employees and other workers, these items are newsworthy: (1) Providing and training the correct use of protective equipment including face masks. (2) Monitoring for strict implementation of health protocols. Therefore, it is necessary to keep training and information up-to-date due to the new phenomena of this disease.

## Data availability statement

The raw data supporting the conclusions of this article will be made available by the authors, without undue reservation.

## Ethics statement

The studies involving human participants were reviewed and approved by Ahvaz Jundishapur University of Medical Sciences (IR.AJUMS.REC.1400.008). The patients/participants provided their written informed consent to participate in this study.

## Author contributions

MS, LIG, and BFD designed the study proposal and questionnaire, analyzed the data, and co-wrote the paper. MS and FH collected the research data. BFD and LIG shared in article writing. All authors contributed to the article and approved the submitted version.

## Conflict of interest

The authors declare that the research was conducted in the absence of any commercial or financial relationships that could be construed as a potential conflict of interest.

## Publisher’s note

All claims expressed in this article are solely those of the authors and do not necessarily represent those of their affiliated organizations, or those of the publisher, the editors and the reviewers. Any product that may be evaluated in this article, or claim that may be made by its manufacturer, is not guaranteed or endorsed by the publisher.
